# Outbreaks Associated with Untreated Recreational Water — United States, 2000–2014

**DOI:** 10.15585/mmwr.mm6725a1

**Published:** 2018-06-29

**Authors:** Daniel S. Graciaa, Jennifer R. Cope, Virginia A. Roberts, Bryanna L. Cikesh, Amy M. Kahler, Marissa Vigar, Elizabeth D. Hilborn, Timothy J. Wade, Lorraine C. Backer, Susan P. Montgomery, W. Evan Secor, Vincent R. Hill, Michael J. Beach, Kathleen E. Fullerton, Jonathan S. Yoder, Michele C. Hlavsa

**Affiliations:** ^1^Department of Family and Preventive Medicine, Emory University School of Medicine, Atlanta, Georgia; ^2^Division of Foodborne, Waterborne, and Environmental Diseases, National Center for Emerging and Zoonotic Infectious Diseases, CDC; ^3^Oak Ridge Institute for Science and Education, Oak Ridge, Tennessee; ^4^Environmental Protection Agency, Washington, DC; ^5^Division of Environmental Hazards and Health Effects, National Center for Environmental Health, CDC; ^6^Division of Parasitic Diseases and Malaria, Center for Global Health, CDC.

Outbreaks associated with untreated recreational water can be caused by pathogens, toxins, or chemicals in fresh water (e.g., lakes, rivers) or marine water (e.g., ocean). During 2000–2014, public health officials from 35 states and Guam voluntarily reported 140 untreated recreational water–associated outbreaks to CDC. These outbreaks resulted in at least 4,958 cases of disease and two deaths. Among the 95 outbreaks with a confirmed infectious etiology, enteric pathogens caused 80 (84%); 21 (22%) were caused by norovirus, 19 (20%) by *Escherichia coli*, 14 (15%) by *Shigella*, and 12 (13%) by *Cryptosporidium*. Investigations of these 95 outbreaks identified 3,125 cases; 2,704 (87%) were caused by enteric pathogens, including 1,459 (47%) by norovirus, 362 (12%) by *Shigella*, 314 (10%) by *Cryptosporidium*, and 155 (5%) by *E. coli*. Avian schistosomes were identified as the cause in 345 (11%) of the 3,125 cases. The two deaths were in persons affected by a single outbreak (two cases) caused by *Naegleria fowleri*. Public parks (50 [36%]) and beaches (45 [32%]) were the leading settings associated with the 140 outbreaks. Overall, the majority of outbreaks started during June–August (113 [81%]); 65 (58%) started in July. Swimmers and parents of young swimmers can take steps to minimize the risk for exposure to pathogens, toxins, and chemicals in untreated recreational water by heeding posted advisories closing the beach to swimming; not swimming in discolored, smelly, foamy, or scummy water; not swimming while sick with diarrhea; and limiting water entering the nose when swimming in warm freshwater.

An outbreak associated with untreated recreational water* is the occurrence of similar illnesses in two or more persons, epidemiologically linked by location and time of exposure to recreational water or to pathogens, toxins, or chemicals aerosolized or volatilized from recreational water into the surrounding air. Public health officials in the 50 states, the District of Columbia, U.S. territories, and Freely Associated States^†^ can voluntarily report recreational water–associated outbreaks to CDC. This report focuses on data on two groups of untreated recreational water–associated outbreaks: 1) those that began during 2000–2012 and were previously reported (*1*), and 2) those that began during 2013–2014 and were electronically reported to the Waterborne Disease and Outbreak Surveillance System (WBDOSS)^§^ by December 31, 2015. Data on each outbreak include case count,^¶^ number of deaths, etiology, setting (e.g., park), and venue (e.g., lake/reservoir/pond) where the exposure occurred, and earliest illness onset date. Poisson regression analysis was conducted to assess the trend in the annual counts of outbreaks.

During 2000–2014, public health officials from 35 states and Guam voluntarily reported 140 untreated recreational water–associated outbreaks that resulted in at least 4,958 cases** (Table) and two deaths. Etiology was confirmed for 103 (74%) outbreaks. Among these, 95 (92%) were caused by pathogens, including five outbreaks with multiple etiologies,^††^ and resulted in at least 3,125 cases; enteric pathogens caused 80 (84%) of the 95 outbreaks and 2,704 (87%) of the 3,125 cases. Among the 95 outbreaks with a confirmed infectious etiology, 21 (22%) were caused by norovirus, 19 (20%) by *E. coli*, 14 (15%) by *Shigella*, and 12 (13%) by *Cryptosporidium*. Investigations of the 95 outbreaks identified 1,459 (47%) cases caused by norovirus, 362 (12%) by *Shigella*, 345 (11%) by avian schistosomes, 314 (10%) by *Cryptosporidium*, and 155 (5%) by *E. coli*. The two deaths occurred within a single outbreak caused by *Naegleria fowleri*.^§§^Of the 103 outbreaks with confirmed etiology, eight (8%) were caused by toxins or chemicals and resulted in at least 78 cases. Of the eight outbreaks caused by toxins or chemicals, seven (88%) were caused by algal toxins from harmful algal blooms.

**TABLE:** Number of untreated recreational water–associated outbreaks, cases, and median number of cases, by etiology —United States, 2000–2014

Etiology	Outbreaks no. (%)	Cases no. (%)	Cases per outbreak median no. (range)
**Total**	140 (100)*	**4,958 (100)**	**9 (2–1,341)**
**Bacterium**	43 (31)	604 (12)	5 (2–141)
*Campylobacter*	1 (1)	6 (0)	6 (—**)^†^**
*Escherichia coli*	19 (14)	155 (3)	5 (3–45)
*Leptospira*	6 (4)	74 (2)	3 (2–43)
*Plesiomonas shigelloides*	3 (2)	7 (0)	2 (2–3)
*Shigella*	14 (10)	362 (7)	14 (2–141)
**Parasite**	25 (18)	685 (14)	7 (2–220)
Avian schistosomes	8 (6)	345 (7)	17.5 (4–200)
*Cryptosporidium*	12 (9)	314 (6)	6.5 (3–220)
*Giardia*	4 (3)	24 (0)	6 (2–10)
*Naegleria fowleri*	1 (1)	2 (0)	2 (—)^†^
**Virus**	22 (16)	1,491 (30)	27.5 (8–597)
Adenovirus	1 (1)	32 (1)	32 (—)^†^
Norovirus	21 (15)	1,459 (29)	26 (8–597)
**Multiple^§^**	5 (4)	345 (7)	56 (45–125)
**Chemical/Toxin**	8 (6)	78 (2)	8.5 (2–20)
Algal toxin	7 (5)	75 (2)	9 (2–20)
Copper sulfate	1 (1)	3 (0)	3 (—)^†^
**Unidentified^¶^**	37 (26)	1,755 (35)**	8 (2–1,341)

* Outbreak etiology proportion by group sums to >100% because of rounding.

^†^ Not applicable because only one outbreak was nationally reported for that etiology.

^§^ The five outbreaks categorized as having multiple etiologies included outbreaks of 1) *Shigella* and *Plesiomonas shigelloides*; 2) *Shigella*, norovirus, and *Yersinia enterolytica*; 3) *Shigella*, *Campylobacter*, and norovirus; 4) *Shigella*, *Escherichia coli*, and *Plesiomonas shigelloides*; and 5) *Giardia* and norovirus.

^¶^ Approximately 1,341 cases were associated with an outbreak with predominantly skin illness caused by an etiology that was unidentified but suspected to be poison ivy when dirt was mixed with water to create an obstacle in an endurance race.

** All outbreaks without a confirmed etiology (e.g., outbreaks with a suspected or unknown etiology) were classified as having an unidentified etiology for this analysis. Unidentified etiology indicates lack of laboratory confirmation but not necessarily absence of traditional epidemiologic and environmental health data indicative of a particular etiology.

Public parks (50 [36%]) and beaches (45 [32%]) were the leading settings associated with the 140 outbreaks. Most outbreaks were associated with a lake/reservoir/pond venue (117 [84%]). Among the 140 outbreaks, the majority started during June–August (113 [81%]), with 65 (58%) staring in July (Figure). None of the outbreaks started during December–February. Poisson regression analyses indicated the annual outbreak count did not change significantly over the 15 years (p = 0.477).

**FIGURE:**
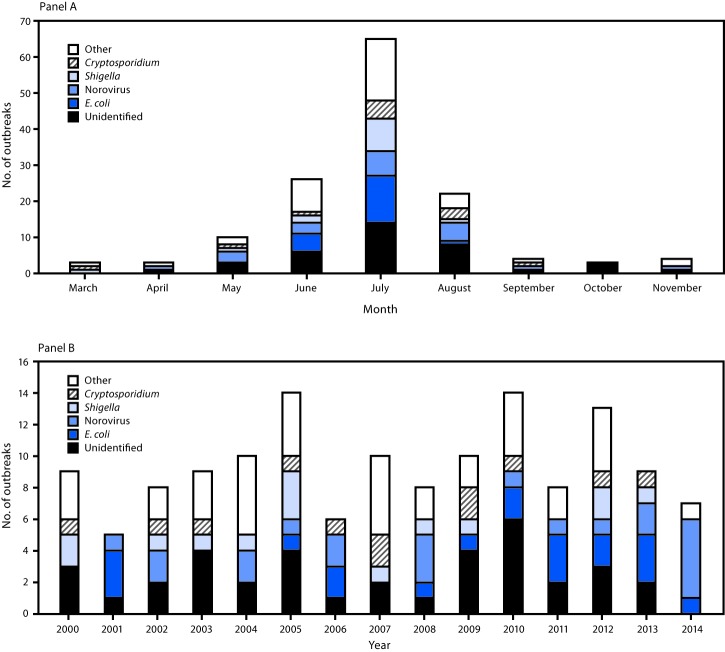
Number* of untreated recreational water–associated outbreaks by etiology and month (panel A) and year (panel B) — United States, 2000–2014^†^ **Abbreviation:** *E. coli* = *Escherichia coli*. * N = 140. ^†^ Other includes all outbreaks of confirmed etiology other than *Cryptosporidium*, *E. coli*, *Shigella*, or norovirus.

## Discussion

A total of 140 untreated recreational water–associated outbreaks were reported to CDC during 2000–2014. The outbreaks of known infectious etiology were caused by a diverse array of chlorine-susceptible pathogens, including enteric bacteria, parasites, and viruses. Many of the pathogens that cause outbreaks in untreated recreational water venues rarely cause outbreaks in treated recreational water (e.g., pools) (*2*). Well-operated, treated recreational water venues in which water disinfectant (chlorine or bromine) concentrations are properly maintained are at decreased risk for pathogen transmission. The diversity among the etiologies of untreated recreational water–associated outbreaks also requires different sets of steps swimmers and parents of young swimmers can take to protect themselves and others from illness.

The untreated recreational water–associated outbreaks were predominantly caused by enteric pathogens. Norovirus, *E. coli*, *Shigella*, *Cryptosporidium*, and other enteric pathogens can be transmitted via untreated recreational water when fecally contaminated water is ingested. Swimmers can be a source of fecal contamination if they have a fecal incident in the water or fecal material washes off their bodies. Other sources of fecal contamination include storm water runoff, flooding, sewage overflows, sewage treatment plant discharges, septic systems, boating waste, and animal waste on or near a beach. *E. coli* and *Cryptosporidium* contamination can be introduced by human or animal feces; norovirus and *Shigella* are indicative of human fecal contamination. Swimming in untreated recreational water that is shallow, poorly circulating, or overcrowded; frequented by children aged <5 years with no or limited toileting skills; without adequate, easily accessible, and well-stocked hygiene facilities (e.g., toilets or diaper-changing stations); or swimming soon after heavy rain can increase risk for exposure to enteric pathogens.

Other etiologies identified in this summary are unique to untreated recreational water. Avian schistosomes can cause cercarial dermatitis (swimmer’s itch) in persons exposed to either freshwater or brackish water in which infected birds contaminate the water and where the intermediate host snails are found. Cercarial dermatitis appears as a skin rash and is caused by an allergic reaction when cercariae in the water penetrate the skin. However, the cercariae do not mature into adult worms in humans, who are accidental hosts.

Algal toxins produced by harmful algal blooms in freshwater or marine water can cause a range of illnesses, from skin or eye irritation to respiratory, gastrointestinal, or neurologic symptoms depending on type of toxin and the route of exposure. In recent years, harmful algal blooms have been observed with increasing frequency and in more locations in the United States, possibly because of increasing nutrient pollution and warming water or improved surveillance (*3*). In 2016, CDC launched the One Health Harmful Algal Bloom System^¶¶^ an electronic system that allows state and territorial public health agencies and their partners to report cases of human or animal illness or environmental data on harmful algal blooms. A better understanding of harmful algal blooms is needed to optimize prevention of associated illness and harmful algal blooms.

*Naegleria fowleri* causes primary amebic meningoencephalitis after water containing the ameba enters the body through the nose and the ameba travels to the brain via the olfactory nerve. Infection, which is usually fatal, typically occurs when persons swim or dive in warm, untreated freshwater. The recent survival of two U.S. patients with primary amebic meningoencephalitis suggests that early diagnosis and treatment might improve outcomes (*4*). Steps can be taken by swimmers and parents of young swimmers to minimize exposure to enteric pathogens, avian schistosomes, algal toxins, and *Naegleria fowleri* in untreated recreational water (Box).

BOX **Preventing exposure to germs and harmful algal bloom toxins in untreated recreational water**

**Stay out of the water if**

- Beach is closed or an advisory is posted for high bacterial levels or other conditions, such as sewage spills or harmful algal blooms.
- A recent heavy rain has occurred.
- A discharge pipe can be seen on the beach.
- Fish or other animals in or near the water are dead.
- Water is discolored, smelly, foamy, or scummy.

**Diarrhea-causing germs**

- Don’t swim or let children swim if sick with diarrhea.
  - If diarrhea is caused by *Cryptosporidium*, wait until 2 weeks after diarrhea has stopped to go swimming.
- Don't swallow recreational swimming water.

https://www.cdc.gov/healthywater/swimming/swimmers/steps-healthy-swimming.html.

**Avian schistosomes**

- Don’t swim near or wade in marshy areas where snails are commonly found.
- Towel dry or shower immediately after exiting the water.

https://www.cdc.gov/parasites/swimmersitch/ .

**Harmful algal blooms**

- Avoid water that contains harmful algal blooms (when in doubt stay out).
- Keep children and pets from drinking discolored, smelly, foamy, or scummy water.
- Get out and rinse off with clean water as soon as possible after swimming in water that might contain a harmful algal bloom.
- Rinse off pets, especially dogs, immediately if they swim in discolored, smelly, foamy, or scummy water. Do not let them lick the algae off their fur.

https://www.cdc.gov/habs/prevention-control.html.

**Naegleria fowleri**

The only certain way to prevent a *Naegleria fowleri* infection caused by swimming is to refrain from water-related activities in warm freshwater. To reduce exposure risk

- Use nose clips, hold your nose shut, or keep head above water when taking part in water-related activities in bodies of warm freshwater.
- Avoid putting your head under the water in hot springs and other untreated thermal waters.
- Avoid water-related activities in warm freshwater during periods of high water temperature.

https://www.cdc.gov/parasites/naegleria/prevention.html.

The findings in this report are subject to at least three limitations. First, the outbreak counts presented likely underestimate actual disease incidence, in part because of variation in public health capacity and reporting requirements across jurisdictions. In addition, untreated recreational water–associated outbreaks might be difficult to detect given that persons who travel long distances to untreated recreational water venues might become ill after returning to geographically dispersed homes in multiple public health jurisdictions, so that the illnesses are never linked to a common exposure (*5*). Entering freshwater and marine water has been associated with a wide range of illnesses despite an absence of reported outbreaks (*5*). Second, for this analysis, all outbreaks without a laboratory-confirmed etiology (e.g., outbreaks with a suspected or unknown etiology) were classified as having an unidentified etiology. Unidentified etiology therefore does not necessarily indicate absence of traditional epidemiologic and environmental health data indicative of a particular etiology. Finally, reporting and review procedures changed over time, which affects the ability to compare data across years.

Given the connections among swimmer health, animal health, and the environment, preventing untreated recreational water–associated outbreaks requires a One Health*** approach. Collaboration among those with expertise across multiple disciplines (including epidemiologists, environmental health practitioners, veterinarians, and ecologists) and multiple sectors working at the human-animal-environment interface should focus on taking steps to prevent and remediate fecal contamination of the water (e.g., prevent sewage overflows and increase water circulation through engineering), manage wildlife (e.g., encourage birds to leave the beach area) and other animals, properly monitor water quality for bacterial concentration and nutrient pollution (which promotes harmful algal blooms), and encourage a robust monitoring and notification program for untreated recreational waters (*6*). Sections of the BEACH Act of 2000^†††^ allow the Environmental Protection Agency to provide grants to coastal and Great Lakes authorities to monitor their beaches and notify the public of potentially unsafe water quality conditions. The related Beach Advisory and Closing Online Notification^§§§^ database provides a resource for swimmers to obtain information on water conditions. However, these are limited to coastal/marine and Great Lakes beaches, whereas most reported outbreaks are associated with smaller, inland lakes, reservoirs, and ponds. This requires swimmers and parents of young swimmers to check for local beach advisories and water conditions in addition to following the steps of healthy swimming. The prevention of untreated recreational water outbreaks includes actions such as engaging and educating the public about healthy swimming, and disseminating healthy swimming messages, particularly before and during June–August. These include heeding posted advisories closing the beach to swimming; not swimming in discolored, smelly, foamy, or scummy water; not swimming while sick with diarrhea; and limiting water entering the nose when swimming in warm freshwater.

**Summary**

**What is already known about this topic?**

Untreated recreational water–associated outbreaks can be caused by pathogens, toxins, or chemicals in freshwater (e.g., lakes) or marine water (e.g., ocean).

**What is added by this report?**

During 2000–2014, 140 untreated recreational water–associated outbreaks that caused at least 4,958 illnesses and two deaths were reported; 80 outbreaks were caused by enteric pathogens.

**What are the implications for public health practice?**

Swimmers should heed posted advisories closing the beach to swimming; not swim in discolored, smelly, foamy, or scummy water; not swim while sick with diarrhea; and limit water entering the nose when swimming in warm freshwater.

## Footnotes

* Untreated recreational water is water that has not undergone a disinfection or treatment process to maintain good microbiological quality for recreation.

† Includes Federated States of Micronesia, Marshall Islands, and Palau.

§ 2013–2014 are the last years for which finalized data were available. For more information on WBDOSS, visit https://www.cdc.gov/healthywater/surveillance/index.html; outbreaks resulting from recreational water exposures on cruise ships are not reported to WBDOSS.

¶ Based on the estimated number of primary cases. For outbreaks that started before 2009, if both the actual and estimated case counts were reported, the estimated case count was used if the population was sampled randomly or the estimated count was calculated by applying the attack rate to a standardized population.

** https://www.cdc.gov/healthywater/surveillance/rec-water-tables-figures.html.

†† The five outbreaks categorized as multiple included outbreaks of 1) *Shigella* and *Plesiomonas shigelloides*; 2) *Shigella*, norovirus, and *Yersinia enterolytica*; 3) *Shigella*, *Campylobacter*, and norovirus; 4) *Shigella*, *Escherichia coli*, and *Plesiomonas shigelloides*; and 5) *Giardia duodenalis* and norovirus.

§§ *Naegleria fowleri* typically causes isolated cases of primary amebic meningoencephalitis. For these two cases, despite an investigation by local public health authorities, the location of common exposure was not definitively identified.

¶¶ https://www.cdc.gov/habs/ohhabs.html.

*** https://www.cdc.gov/onehealth.

††† Coastal Recreation Water Quality Monitoring, 33 U.S.C. Sect 1346 (2006). https://www.gpo.gov/fdsys/pkg/USCODE-2011-title33/pdf/USCODE-2011-title33-chap26-subchapIV-sec1346.pdf; Report on Coastal Recreation Waters, 33 U.S.C Section 1375a (2000). https://www.gpo.gov/fdsys/pkg/USCODE-2010-title33/pdf/USCODE-2010-title33-chap26-subchapV-sec1375a.pdf.

§§§ https://watersgeo.epa.gov/beacon2/.
